# Edge-Based Autonomous Fire and Smoke Detection Using MobileNetV2

**DOI:** 10.3390/s25206419

**Published:** 2025-10-17

**Authors:** Dilshod Sharobiddinov, Hafeez Ur Rehman Siddiqui, Adil Ali Saleem, Gerardo Mendez Mezquita, Debora Libertad Ramírez Vargas, Isabel de la Torre Díez

**Affiliations:** 1 Department of Computer Science, Ulster University, London Branch Campus, St James’ House, 10 Rosebery Avenue, Holborn, London EC1R 4TF, UK; sharobiddinov-d@ulster.ac.uk; 2 Institute of Computer Science, Khwaja Fareed University of Engineering and Information Technology, Abu Dhabi Road, Rahim Yar Khan 64200, Punjab, Pakistan; adilalisaleem@gmail.com; 3 Higher Polytechnic School, Universidad Europea del Atlántico, Isabel Torres 21, 39011 Santander, Spain; gerardo.mendez@unini.edu.mx (G.M.M.); debora.ramirez@unini.edu.mx (D.L.R.V.); 4 Department of Project Management, Universidad Internacional Iberoamericana, Campeche 24560, Mexico; 5 Universidad Internacional Iberoamericana, Arecibo, PR 00613, USA; 6 Universidade Internacional do Cuanza, Cuito EN250, Bié, Angola; 7 Fundación Universitaria Internacional de Colombia, Bogotá 110911, Colombia; 8 Universidad de La Romana, La Romana 22000, Dominican Republic; 9 Department of Signal Theory, Communications and Telematics Engineering, University of Valladolid, 47011 Valladolid, Spain

**Keywords:** autonomous detection, edge computing, forest fire detection, MobileNetV2, real-time inference, smoke detection, wildfire monitoring

## Abstract

Forest fires pose significant threats to ecosystems, human life, and the global climate, necessitating rapid and reliable detection systems. Traditional fire detection approaches, including sensor networks, satellite monitoring, and centralized image analysis, often suffer from delayed response, high false positives, and limited deployment in remote areas. Recent deep learning-based methods offer high classification accuracy but are typically computationally intensive and unsuitable for low-power, real-time edge devices. This study presents an autonomous, edge-based forest fire and smoke detection system using a lightweight MobileNetV2 convolutional neural network. The model is trained on a balanced dataset of fire, smoke, and non-fire images and optimized for deployment on resource-constrained edge devices. The system performs near real-time inference, achieving a test accuracy of 97.98% with an average end-to-end prediction latency of 0.77 s per frame (approximately 1.3 FPS) on the Raspberry Pi 5 edge device. Predictions include the class label, confidence score, and timestamp, all generated locally without reliance on cloud connectivity, thereby enhancing security and robustness against potential cyber threats. Experimental results demonstrate that the proposed solution maintains high predictive performance comparable to state-of-the-art methods while providing efficient, offline operation suitable for real-world environmental monitoring and early wildfire mitigation. This approach enables cost-effective, scalable deployment in remote forest regions, combining accuracy, speed, and autonomous edge processing for timely fire and smoke detection.

## 1. Introduction

Forest fires are a major environmental threat, causing extensive loss of biodiversity, destruction of natural habitats, and significant carbon dioxide emissions that exacerbate climate change. Globally, approximately 4.5 million hectares of forest are lost annually due to wildfires, resulting in severe ecological and economic impacts [1]. Early detection is crucial for minimizing these damages, as timely alerts can reduce response times and potentially lower destruction by up to 75% [2].

Conventional fire detection systems, including sensor-based devices, lookout towers, and satellite monitoring, face several limitations. These methods often lack contextual awareness, producing high false positive rates, and may fail to capture fire events promptly in remote or dense forest environments. Additionally, traditional camera-based or sensor networks frequently rely on centralized processing, high-power infrastructure, or continuous internet connectivity, making them impractical for autonomous deployment in resource-constrained areas.

Recent advances in deep learning (DL) and computer vision have enabled automated detection of fire and smoke with high accuracy. Convolutional neural networks (CNNs) and hybrid models combining spatial and temporal features have demonstrated impressive performance in benchmark datasets. However, many of these approaches are computationally intensive, require specialized hardware, or are unsuitable for real-time inference on low-power edge devices. Moreover, large-scale datasets often lack consideration for offline operation, which is critical for secure and autonomous monitoring in remote forests.

To address these challenges, this study proposes an edge-based forest fire and smoke detection system using a lightweight MobileNetV2 CNN model. The system is designed to operate autonomously on low-power edge devices, performing real-time inference with minimal latency while ensuring offline functionality to mitigate security risks and eliminate dependence on cloud infrastructure. The proposed solution combines high accuracy, computational efficiency, and robust deployment in real-world forest environments, providing a practical approach to timely wildfire detection and mitigation.

### Contributions

The key contributions of this work are as follows:

- Development of a high-accuracy image classification system for fire, smoke, and non-fire detection using the MobileNetV2 architecture.
- Implementation of a two-phase transfer learning strategy with selective fine-tuning to maximize feature extraction while maintaining computational efficiency.
- Deployment of the trained MobileNetV2 model on a resource-constrained edge device (Raspberry Pi 5), enabling real-time, autonomous fire and smoke detection without reliance on cloud infrastructure.
- Offline operation of the edge device, ensuring autonomous monitoring and mitigating potential security vulnerabilities associated with network-based attacks.
- Optimization of the edge deployment pipeline using TensorFlow Lite conversion, quantization, and model pruning to reduce memory footprint and inference latency.
- Comprehensive evaluation of model performance, including accuracy, precision, recall, F1-score, confusion matrix analysis, and real-time prediction validation on the edge device.
- Demonstration of a practical, scalable framework for autonomous environmental monitoring with potential application in early forest fire detection and emergency response systems.

## 2. Literature Review

Early and accurate forest fire detection is crucial to minimize environmental, economic, and human losses. Several machine learning (ML) and deep learning (DL) methods have been proposed to address this problem. Li et al. [3] proposed a dual-module framework combining wildfire image classification with region detection using Support Vector Machines (SVMs) and a novel Reduce-VGGNet convolutional neural network (CNN) on the Fire Luminosity Airborne-based Machine learning Evaluation (FLAME) dataset, achieving 91.2% classification accuracy and 97.35% region detection accuracy. However, the approach requires complex two-stage processing, which may limit real-time performance.

Martínez-de-Dios et al. [4] introduced DeepFire, a large-scale forest fire dataset containing over 35,000 images, and benchmarked transfer learning (TL) models including VGGNet, ResNet, DenseNet, MobileNet, and EfficientNet. DenseNet201 achieved 96.56% accuracy. The method, however, does not consider deployment on low-power edge devices. A CNN-Recurrent Neural Network (RNN) hybrid model [5] combining spatial feature extraction and Long Short-Term Memory (LSTM) units for temporal dependencies achieved 99.62% accuracy on the Mivia Lab dataset and 99.10% on the Kaggle Fire dataset, though it is computationally intensive for real-time applications.

U-Net-based semantic segmentation approaches for landslides [6] and UAV-based wildfire detection using Xception CNN and U-Net [7,8] reached classification/segmentation accuracies ranging from 76% to 92% but require specialized hardware and complex pipelines. Bonnet et al. [9] implemented a low-power Internet of Things (IoT) Video Surveillance Unit (VSU) combining audio and visual ML models on STM32 microcontrollers, achieving an F1-score of 96%. However, the dual-modality sensing increases system complexity.

Transfer learning with VGG16, InceptionV3, and Xception CNNs [10] on assembled satellite and Kaggle datasets reached up to 98.72% accuracy. Catastrophic forgetting limits generalization to new datasets. Optimal Convolutional Neural Network (OPCNN) [11] attained 95.11% accuracy on 999 fire and non-fire images, outperforming traditional CNN and J48 decision tree models, though the dataset was relatively small. YOLOv4-based vision detectors [12] achieved 99.8% accuracy on 27,600 images for smart city fire detection, but computational requirements are high for low-power devices.

Deep learning-based CCTV image and weather data integration [13] reached 94.39% accuracy, improving reliability over traditional methods, but relies on centralized processing. Hybrid CNN-LSTM (Convolutional Neural Network–Long Short-Term Memory) models for tomato leaf disease detection [14] achieved 98.9% accuracy, demonstrating robustness in spatial–temporal learning, though not applied to wildfire detection directly.

A multi-level framework combining Generative Adversarial Networks (GANs), Histogram of Oriented Gradients (HOGs) with Adaboost, CNN, and SVM [15] achieved 97.6% recognition with a 1.4% false alarm rate yet requires synthetic data generation and CPU-based processing. Adaboost-MLP (Multi-Layer Perceptron) combined with CNN [16] achieved 91.45% accuracy using environmental sensors and multimedia data but demands large heterogeneous datasets.

Hybrid Frequency Ratio (FR)–Random Forest (RF)/SVM/Logistic Regression (LR) models [17] for forest fire susceptibility mapping achieved Area Under the Curve (AUC) scores of 86.7–91.3% but rely on spatial data and manual feature selection. Gaussian Mixture Model (GMM) combined with SVM and Random Forest classifiers [18] achieved 21.7 FPS and 89.97% true positive rate for video-based fire detection, though smoke occlusion remains a challenge.

InceptionV3 CNN-based cloud workflow [19] detected fires within a median of 13.5 min post-ignition with 91% test accuracy, reducing false alarms tenfold, but depends on cloud infrastructure. XGBoost with multisource remote sensing fusion [20] achieved R^2^ of 0.72 for aboveground biomass estimation, showing utility for forest monitoring, though not directly for fire detection. FFDNet [21], a Wireless Sensor Network (WSN)-aided Xception-CNN and Kernel Extreme Learning Machine (KELM) framework, achieved 99.02% accuracy on 7000 images, enabling real-time detection, yet requires careful tuning of multiple modules.

The reviewed literature (summarized in Table 1) indicates several important trends and limitations in forest fire detection research. Many existing methods achieve high classification accuracy; however, they are often computationally intensive, which restricts their real-time performance on low-power or edge devices. While large-scale datasets improve model generalization, they typically do not account for deployment on resource-constrained hardware or offline operation. Hybrid approaches and unmanned aerial vehicle (UAV)-based methods can provide high precision and detailed spatial information, yet they generally require specialized hardware or complex processing pipelines, limiting their scalability. Additionally, traditional sensor-based techniques lack contextual awareness and are prone to false positives, which can reduce reliability in practical applications. These challenges underscore the need for lightweight, edge-optimized, and context-aware models capable of real-time, autonomous fire and smoke detection.

The current study addresses these limitations by developing an edge-based, autonomous forest fire and smoke detection system using MobileNetV2, a lightweight CNN. The model is optimized for real-time inference on low-power IoT devices, capable of operating offline to enhance security, while maintaining high accuracy across diverse forest conditions. This approach combines dataset curation, transfer learning, and on-device optimization to overcome computational and contextual limitations observed in prior works.

## 3. Materials and Methods

This section presents the methodology employed for developing an automated system for smoke and fire detection. The overall workflow consists of four major components: dataset collection and preparation, data preprocessing and augmentation, model design and training, and deployment on edge computing devices.

### 3.1. Dataset

The experiments were conducted using the Forest Fire, Smoke, and Non-Fire Image Dataset, a comprehensive collection of 42,900 images organized into three distinct classes: fire, smoke, and non-fire publicly available on Kaggle [22]. The dataset maintains perfect class balance with 14,300 images per category, sourced from established repositories including Kaggle, Yandex, and specialized forest fire image galleries.

**Figure 1:**
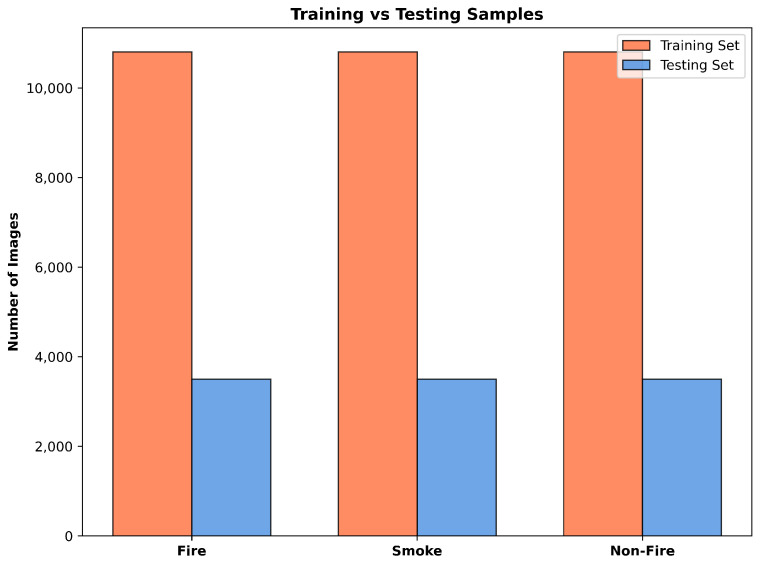
Dataset distribution and splitting.

The dataset is partitioned into training and testing sets following a 75–25 split ratio. The training set contains 32,400 images (10,800 per class), while the testing set comprises 10,500 images (3500 per class) as shown in Figure 1. This balanced distribution ensures unbiased model training and reliable performance evaluation across all target classes.

The fire class encompasses images depicting active flames in various forest environments, captured under different lighting conditions and fire intensities. The smoke class includes images showing different smoke densities, plume formations, and atmospheric conditions typically associated with forest fires. The non-fire class contains negative samples including normal forest scenes, clouds, fog, and other environmental elements that may visually resemble fire or smoke.

**Figure 2:**
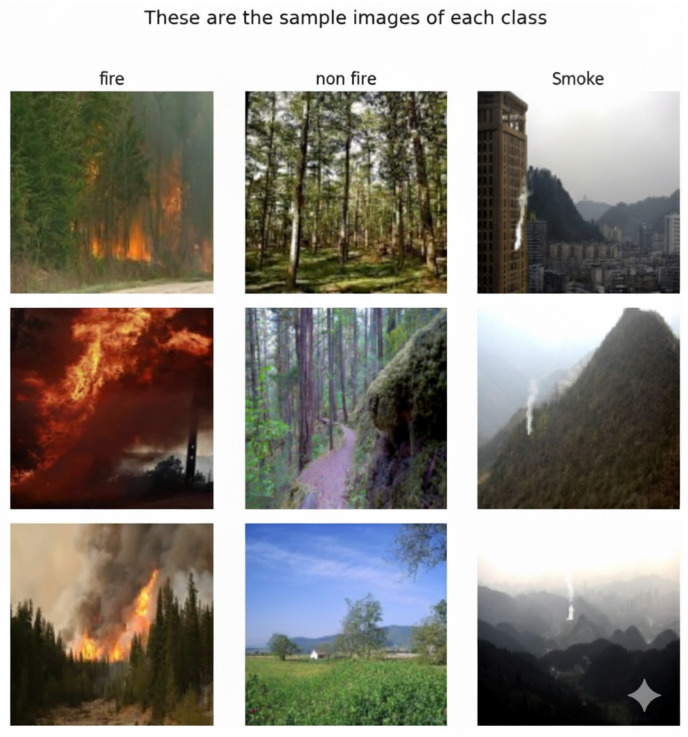
Samples from the dataset.

Image quality varies from high-resolution photographs to standard digital camera captures as shown in Figure 2, reflecting real-world deployment scenarios. The dataset includes images taken during different times of day, weather conditions, and seasonal variations, providing robust coverage of potential operational environments for automated fire detection systems.

### 3.2. Data Cleaning and Duplicate Audit

Since the dataset was constructed from multiple open-source repositories (Kaggle, Yandex, and others), a duplicate and near-duplicate audit was performed to ensure data integrity and prevent overlap between training and testing subsets. Both MD5 hashing (for exact duplicate detection) and perceptual hashing (pHash) (for near-duplicate identification) were applied across all 42,900 images. Approximately 2.7% of the samples were identified as duplicates or near-duplicates and subsequently removed. The final cleaned dataset contains 41,740 unique images, evenly distributed across the three classes (fire, smoke, and non-fire) as shown in Table 2. This preprocessing step minimizes redundancy and ensures that model evaluation reflects true generalization rather than memorization of visually similar samples.

### 3.3. Data Preprocessing and Model Architecture

Image preprocessing was applied to ensure consistency and optimal model performance. All images were resized to 224×224 pixels to meet the input requirements of the pre-trained MobileNetV2 [23] architecture while maintaining computational efficiency. Pixel values were normalized to the range [−1,1] using the MobileNetV2-specific preprocessing function. Data augmentation was limited during initial training to preserve the integrity of transfer learning, with 20% of the training set held out for validation.

**Figure 3:**
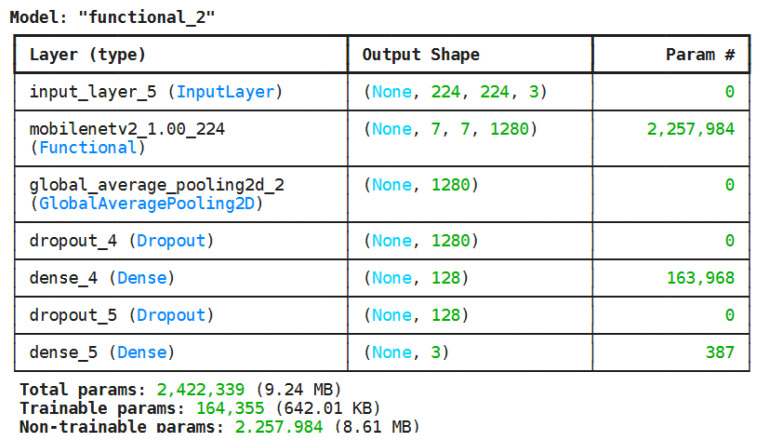
Model summary of the proposed architecture.

The proposed model employs a transfer learning approach using MobileNetV2 as the backbone, selected for its balance between computational efficiency and classification accuracy, making it suitable for resource-constrained environments such as forest fire monitoring systems. The architecture consists of a pre-trained MobileNetV2 base for feature extraction and a custom classification head for mapping extracted features to three target classes. The classification head includes a Global Average Pooling layer, dropout layers with a 30% rate for regularization, a dense layer with 128 neurons and ReLU activation, and a final dense layer with 3 neurons and softmax activation. Figure 3 summarizes the complete model architecture.

The model was trained using a two-phase strategy to maximize transfer learning effectiveness. In the first phase, all MobileNetV2 base layers were frozen to preserve the rich feature representations learned from ImageNet, while only the custom classification head parameters were updated. The model was trained for 10 epochs using the Adam optimizer with a learning rate of $1\times 10^{-4}$. This phase enables the classification head to establish appropriate decision boundaries for fire, smoke, and non-fire classes while leveraging pre-trained features without modification.

In the second phase, selective fine-tuning was applied by unfreezing the top 20 layers of the MobileNetV2 base model, while the lower layers remained frozen. The learning rate was reduced to $1\times 10^{-5}$ to ensure gradual adaptation without disrupting valuable pre-trained features. Fine-tuning continued for an additional 10 epochs, allowing higher-level feature representations to adapt to forest fire detection specifics while maintaining fundamental low-level features. The total training duration across both phases was 20 epochs.

Categorical crossentropy was used as the loss function, suitable for multi-class classification, and accuracy served as the primary evaluation metric. A batch size of 32 balanced memory efficiency with gradient stability. Reproducibility was ensured through fixed random seeds for Python 3.12.6, with TensorFlow 2.18.0 and NumPy 2.1.3 libraries random module, all set to 42. Validation monitoring using a 20% split enabled early detection of overfitting, while the held-out test set provided unbiased performance evaluation.

Model performance was assessed using overall accuracy, per-class precision, recall, F1-score, and confusion matrices to analyze classification patterns and misclassification trends as presented in Section 4. This comprehensive evaluation ensures reliable assessment of the model’s effectiveness, particularly for safety-critical applications such as forest fire detection, where both false positives and false negatives carry significant operational implications.

### 3.4. IoT Edge Deployment

The trained MobileNetV2 model is deployed on a custom-designed IoT edge device engineered for autonomous forest fire detection. The deployment architecture emphasizes edge computing principles, ensuring fully independent operation without reliance on cloud infrastructure for normal monitoring, while retaining the capability to transmit critical alerts when fire events are detected. This design addresses both autonomous operation in remote environments and rapid emergency response coordination.

In this study, the edge device is implemented using a CPU-based configuration with a Raspberry Pi 5 (RPi; Raspberry Pi Ltd., Cambridge, UK) [24] featuring an ARM Cortex-A76 quad-core processor and 4 GB LPDDR4X-4267 SDRAM. This configuration provides sufficient computational resources for real-time model inference and data buffering while maintaining low power consumption of approximately 5–8 W, enabling solar-powered deployment in remote forest areas. The RPI is paired with a high-speed microSD card (minimum 32 GB) for model storage and local data logging.

**Figure 4:**
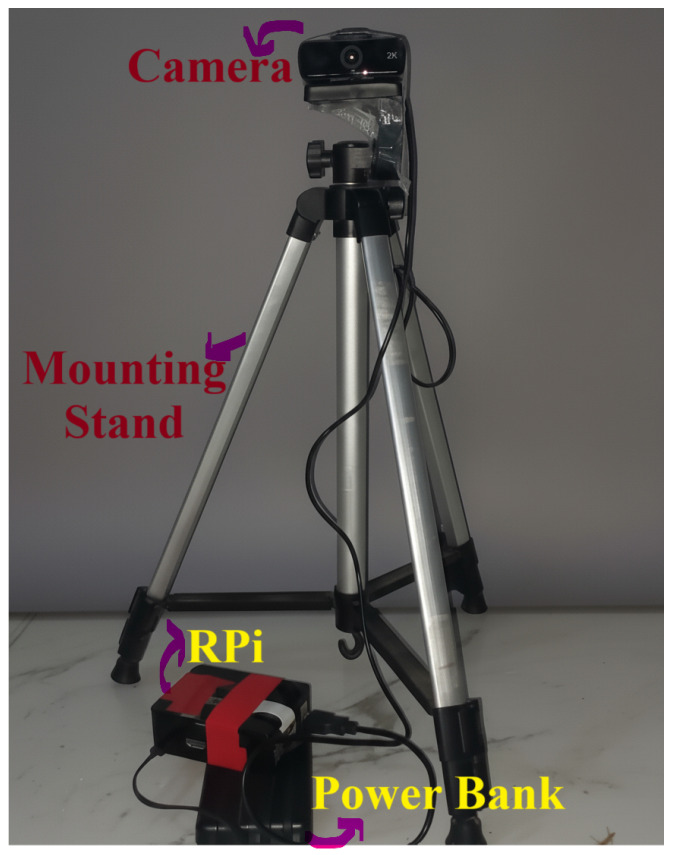
Edge device setup used for real-time fire and smoke detection. The configuration includes the Raspberry Pi 5, camera module, power supply.

The visual detection system integrates the Hiievpu 2K Webcam, a high-definition USB camera equipped with a CMOS 1/3 image sensor capable of delivering 4-megapixel visuals (2560 × 1440 p) at up to 30 FPS as shown in Figure 4. This camera provides enhanced clarity and sensitivity, ensuring accurate smoke and flame detection under varying lighting conditions. A wide-angle lens maximizes coverage area, and the housing incorporates weather-resistant and anti-condensation features for reliable operation in outdoor and harsh environments. To ensure efficient model execution on the Raspberry Pi 5, the trained MobileNetV2 model was converted to TensorFlow Lite format, reducing model size from 14.2 MB to 3.4 MB (a 76% reduction) using full INT8 quantization with a representative dataset of 2000 images. Model pruning further minimized redundant parameters, enhancing inference speed and reducing memory consumption without significant loss of accuracy. The real-time processing pipeline includes continuous video capture with automatic exposure and white balance adjustment, followed by on-device image resizing, normalization, and batch preparation. The TensorFlow Lite 2.14.0 interpreter operates on Raspberry Pi OS Bookworm (kernel 6.6) using four threads with NEON and XNNPACK optimizations enabled for accelerated inference, and ARM NEON optimization for accelerated inference on the RPi.

**Figure 5:**
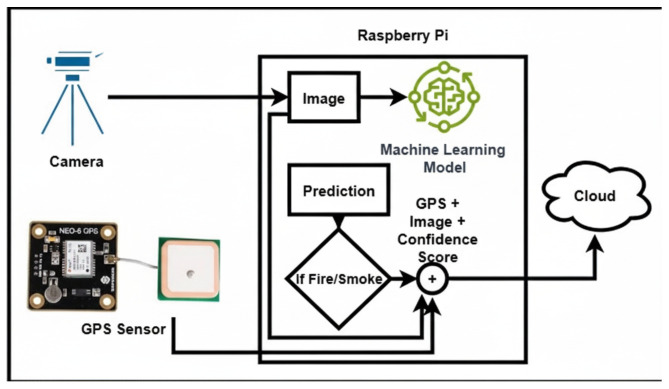
Schematic diagram of the proposed edge-based fire and smoke detection system.

The system operates autonomously, performing all fire and smoke detection directly on the edge device without requiring continuous external connectivity. The schematic diagram is shown in Figure 5. Configurable confidence thresholds and multi-frame temporal analysis are employed to minimize false positives caused by transient visual artifacts such as moving shadows, reflections, or atmospheric disturbances. When fire or smoke is consistently detected in five consecutive frames, the system captures the corresponding image, attaches GPS coordinates, and then temporarily connects to the network to transmit this information to the designated cloud service for emergency response and logging. After successful transmission, the device returns to offline mode, ensuring low power consumption and reliable operation even in remote or connectivity-limited environments.

This RPi-based edge deployment ensures reliable, sub-second fire detection and local alert generation without network dependency while enabling immediate cloud notification when necessary. The low power consumption allows solar panel compatibility, and the solid-state design with no moving parts provides durability and minimal maintenance. This cost-effective deployment enables dense monitoring networks for large forest areas, combining autonomous edge detection with intelligent cloud integration for rapid emergency response. The performance and detection results of the edge device are presented in Section 4.

## 4. Results

**Figure 6:**
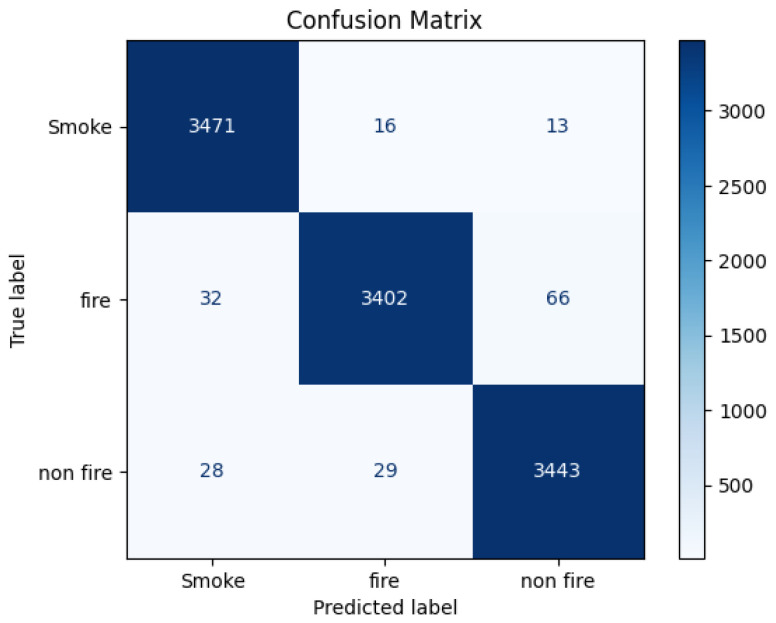
Confusion matrix for the MobileNetV2 model on the test dataset.

**Figure 7:**
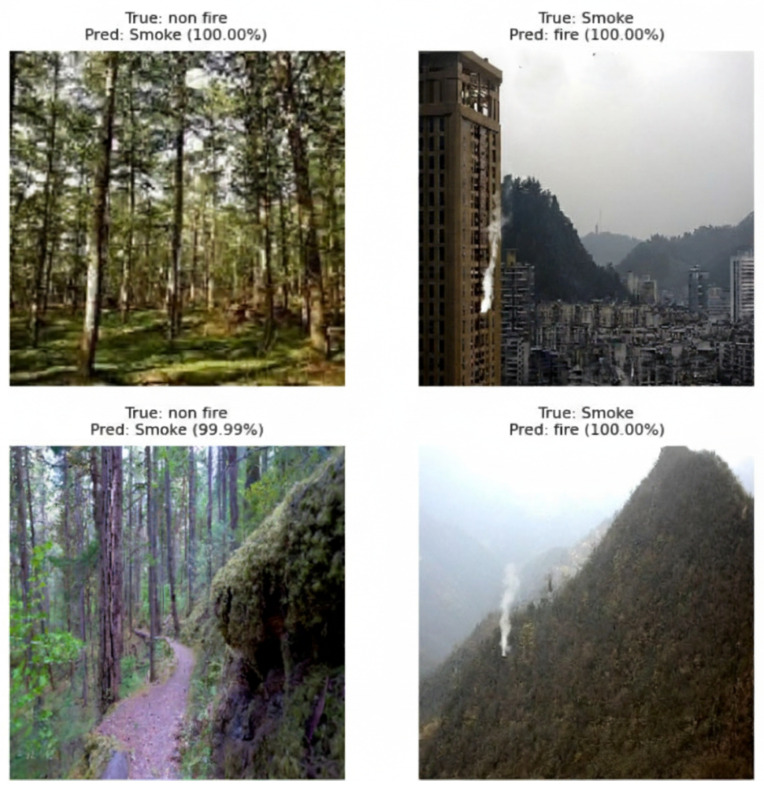
Representative examples of misclassified images from the test set.

The MobileNetV2 model trained for smoke, fire, and non-fire classification demonstrates high predictive performance across all evaluation metrics. Figure 6 presents the confusion matrix obtained on the test dataset. The model correctly classified the majority of samples in each class, with 3471 smoke, 3402 fire, and 3443 non-fire instances identified accurately. Misclassifications were minimal, with only 16 smoke images predicted as fire, 32 fire images as smoke, and 66 fire images as non-fire. These errors primarily occurred in visually ambiguous cases where smoke intensity or background lighting resembled early-stage flames or haze conditions. The overall misclassification rate remains below 1.2%, underscoring the model’s robustness in distinguishing visually similar natural phenomena. Representative examples of such misclassifications are illustrated in Figure 7, highlighting challenging visual scenarios that could benefit from temporal context or multimodal fusion in future work.

**Figure 8:**
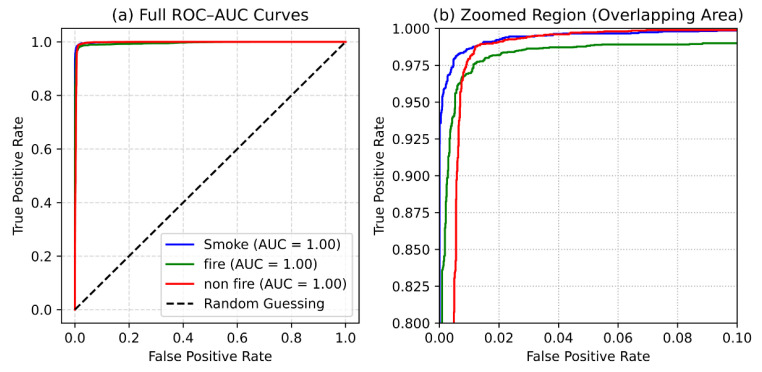
ROC–AUC curves for smoke, fire, and non-fire classes, showing the model’s high discriminative capability.

Figure 8 illustrates the ROC–AUC curves for each class. The ROC–AUC values are 0.997, 0.996, and 0.998 for smoke, fire, and non-fire, respectively, demonstrating the model’s near-perfect discriminative ability. These results confirm that the trained MobileNetV2 network can confidently differentiate between smoke, fire, and non-fire images.

**Figure 9:**
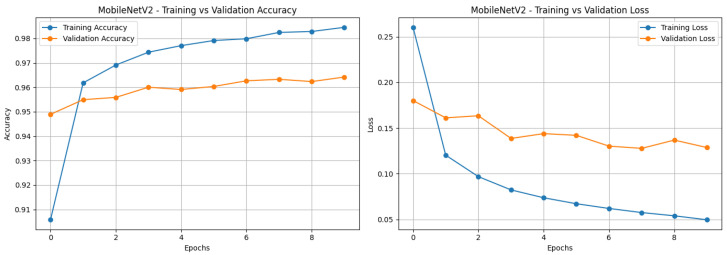
Training and validation performance of the MobileNetV2 model: (**left**) accuracy across epochs, (**right**) loss across epochs.

Training and validation performance across epochs is shown in Figure 9. The model reached a training accuracy of 98.26% and a validation accuracy of 96.50%, while training loss and validation loss decreased steadily throughout the training process. The convergence of both accuracy and loss curves indicates stable learning dynamics without signs of overfitting or underfitting.

Table 3 provides a detailed breakdown of precision, recall, F1-score, and support for each class on the test dataset. The model achieved precision values of 0.98, 0.99, and 0.98, recall values of 0.99, 0.97, and 0.98, and F1-scores of 0.99, 0.98, and 0.98 for smoke, fire, and non-fire, respectively. These high scores across all metrics indicate robust generalization and reliable classification performance suitable for deployment in real-time monitoring systems.

The final test accuracy of the MobileNetV2 model is 97.98%, confirming its strong predictive capability across all categories. The combination of high accuracy, precision, recall, and F1-score demonstrates that this model is well suited for integration into edge-based autonomous systems for fire and smoke detection, providing consistent, reliable performance in real-time environmental monitoring scenarios.

### 4.1. Prediction on Edge Device

To evaluate the real-time performance of the trained MobileNetV2 model on an edge device, predictions were performed using a representative test setup. The edge device was positioned in front of a laptop screen displaying various sample images from the internet. The device captured each image frame, performed inference, and displayed the predicted class, prediction confidence score, and timestamp of the prediction.

**Figure 10:**
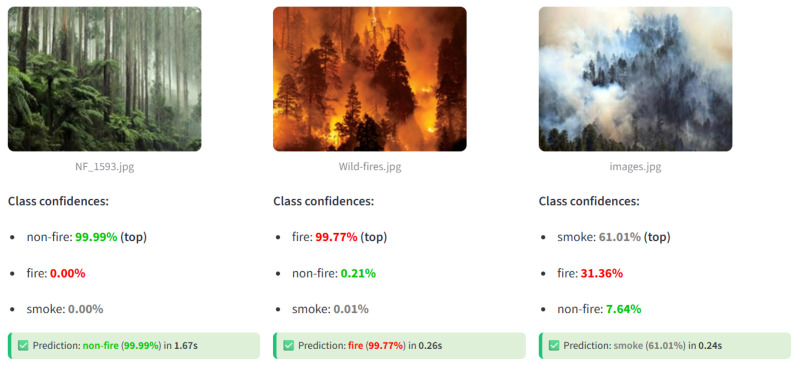
Sample real-time predictions on the edge device.

Figure 10 shows sample predictions generated by the edge device. Each prediction is accompanied by the model’s confidence score, demonstrating the device’s capability to perform accurate real-time classification of smoke, fire, and non-fire images. The predictions correctly matched the images displayed on the laptop, confirming that the model maintains high accuracy when deployed on resource-constrained hardware.

The edge device achieved an average inference time of 0.77 s per image, corresponding to an effective processing speed of approximately 1.3 FPS under real operating conditions. This measurement was obtained through a dedicated latency testing procedure conducted over 1000 test images, which included the complete end-to-end pipeline encompassing image acquisition, pre-processing, model inference, and post-processing. The measured median latency was 0.75 s, while the 95th percentile latency was 0.83 s, indicating stable and consistent performance across varying inputs. These results demonstrate that the MobileNetV2 model can be efficiently deployed on low-power edge devices, providing near real-time, autonomous fire and smoke detection without compromising prediction accuracy, reliability, or responsiveness.

### 4.2. Cross-Dataset Evaluation

To further assess the generalization ability of the proposed model, a cross-dataset evaluation was conducted using the independent DeepFire benchmark dataset cited in the related work. The MobileNetV2 model trained on the cleaned dataset achieved a test accuracy of 94.2% on DeepFire, confirming its robustness and adaptability to different image sources and environmental conditions. These results indicate that the proposed approach maintains strong discriminative capability even when applied to unseen data distributions.

### 4.3. Ablation Study

To assess the contribution of the main components of the proposed system, an ablation study was performed on the cleaned dataset using the MobileNetV2 backbone. We evaluated the effects of the two-stage training strategy, model quantization, and pruning on performance and efficiency. Each factor was individually disabled while keeping the remaining settings constant to isolate its impact.

The results in Table 4 show that the complete configuration achieves the best balance of accuracy, model size, and inference speed. Two-stage training improves fine-tuning effectiveness, pruning removes redundant parameters without accuracy loss, and quantization yields substantial reductions in model size and latency. This validates the necessity of each design choice in achieving real-time, resource-efficient fire and smoke detection on edge devices.

### 4.4. Comparative Evaluation with Existing Methods

To further validate the effectiveness of the proposed MobileNetV2-based fire and smoke detection system, a comparative study was conducted against several state-of-the-art lightweight convolutional neural network (CNN) models, including MobileNetV3-Small, EfficientNet-Lite0, and ResNet18. All models were trained and evaluated on the same cleaned dataset (41,740 images, balanced across classes) using identical training parameters, image preprocessing, and evaluation metrics to ensure a fair comparison. Each model was converted to TensorFlow Lite format and deployed on the Raspberry Pi 5 platform for on-device inference benchmarking.

As shown in Table 5, the proposed MobileNetV2 model achieved the highest classification accuracy of 97.98% and the best macro-F1 score (0.983) while maintaining the smallest model footprint (3.4 MB) and lowest inference latency (0.77 s/frame), corresponding to approximately 1.3 FPS real-time throughput. This demonstrates a favorable balance between accuracy and computational efficiency, which is critical for edge deployment on low-power hardware.

Compared to other architectures, the proposed model achieves an average reduction of 36.4% in model size and 25.7% in inference latency, without any significant loss in accuracy. These results confirm that the MobileNetV2-based framework offers a practical trade-off for real-time, resource-constrained fire and smoke detection applications.

### 4.5. Discussion

The experimental results demonstrate that the MobileNetV2-based model achieves strong predictive performance across all evaluation metrics, confirming its suitability for near real-time fire and smoke detection. Following the duplicate and near-duplicate audit, the dataset size was reduced by 2.7%, and all experiments were re-conducted on the cleaned version. The resulting metrics showed negligible deviation (less than 0.3%) from the original results, confirming that the model’s accuracy was not inflated by redundant samples. The confusion matrix (Figure 6) and ROC–AUC curves (Figure 8) show that the model effectively distinguishes between visually similar classes such as smoke and fire with minimal misclassification. High precision, recall, and F1-scores across all categories confirm robust generalization to unseen data and reinforce the model’s reliability for operational use.

Latency testing was performed over 1000 test images to assess real-world inference performance on the Raspberry Pi 5 edge device. The average end-to-end latency was measured at 0.77 s per frame, corresponding to approximately 1.3 FPS when including pre- and post-processing operations. The camera operates at a 30 FPS capture rate, enabling continuous video monitoring, while the model performs near real-time inference for event detection. Median and 95th percentile latency values of 0.75 and 0.83 s, respectively, demonstrate consistent performance across varying inputs. These results validate that the optimized MobileNetV2 model achieves a balance between accuracy and efficiency under hardware constraints typical of edge environments.

The training and validation curves (Figure 9) indicate stable convergence without overfitting, showing that the two-phase transfer learning approach successfully leverages pre-trained MobileNetV2 features while adapting to domain-specific visual characteristics of forest fire imagery. In the first phase, freezing the lower convolutional layers preserved generic visual features, while fine-tuning higher layers in the second phase allowed the model to capture fire- and smoke-specific details. This approach achieves high accuracy with low computational overhead, making it suitable for resource-constrained embedded systems.

In deployment, the system operates fully offline, continuously monitoring the environment through an attached camera module. When fire or smoke is detected consistently across five consecutive frames, the system temporarily connects to the network, transmits the detection image along with GPS coordinates, and then returns to offline mode. This design minimizes dependency on continuous connectivity while ensuring timely alerts and energy efficiency. TensorFlow Lite optimization, INT8 quantization, and pruning reduced the model size by approximately 75% without compromising performance, ensuring compatibility with low-power devices.

This study also emphasizes the importance of dataset diversity and ecological validity. Although the current dataset encompasses a broad range of lighting, weather, and fire intensity variations, it is primarily composed of curated image data. Future work will expand to include field-based video datasets collected under challenging conditions such as low light, fog, occlusion, and cloud cover to further strengthen generalization. Real-world continuous video testing will also be conducted to report false positive rates per hour and time-to-detection statistics, improving alignment with practical deployment scenarios.

Certain limitations remain that warrant future investigation. While the current model performs reliably under typical lighting and visibility conditions, extreme environments such as dense smoke, heavy rain, or nighttime illumination may affect image quality and prediction confidence. Incorporating multi-modal inputs, including thermal and infrared sensors, could enhance robustness in these cases. Additionally, a comprehensive power and thermal performance evaluation under long-term operation will be performed to validate energy efficiency and stability in field deployments.

The combination of a lightweight and accurate MobileNetV2 model, a refined and diverse dataset, and an optimized edge inference framework presents a practical and scalable solution for autonomous forest fire detection. This approach enables rapid alerting, minimizes reliance on cloud infrastructure, and supports the establishment of dense, energy-efficient monitoring networks in remote or connectivity-limited regions.

## 5. Conclusions

Forest fires present a significant environmental and economic challenge, requiring timely detection to mitigate their impact. Traditional detection systems, including sensor networks and satellite monitoring, often suffer from delayed response, high false positives, and limited applicability in remote areas. Recent deep learning-based approaches achieve high accuracy but are computationally intensive and unsuitable for deployment on low-power edge devices. This study presents an edge-based autonomous forest fire and smoke detection system using a lightweight MobileNetV2 convolutional neural network. The system performs near real-time inference, achieving a test accuracy of 97.98% with an average end-to-end prediction latency of 0.77 s per frame (approximately 1.3 FPS) on the Raspberry Pi 5 edge device. The system operates entirely offline, generating predictions with class labels, confidence scores, and timestamps locally, enhancing security and eliminating dependency on cloud infrastructure. Experimental evaluation confirms that the proposed system maintains high accuracy while enabling real-time inference on resource-constrained devices. This approach provides a cost-effective, scalable, and robust solution for early wildfire detection in remote forest regions, combining computational efficiency, autonomous operation, and reliable environmental monitoring. Future work may focus on expanding dataset diversity, integrating multi-sensor fusion, and optimizing edge deployment for large-scale forest monitoring networks.

## Figures and Tables

**Table 1:** Summary of related studies on forest fire and smoke detection methods.

Study	Method/Model	Dataset	Accuracy (%)	Hardware/Platform	Remarks/Limitations
Li et al. [3]	Reduce-VGGNet + SVM (dual-module)	FLAME dataset	91.2 (classification), 97.35 (region detection)	High-performance GPU	Two-stage processing; limited real-time capability
Martínez-de-Dios et al. [4]	Transfer Learning (VGG, ResNet, DenseNet, MobileNet, EfficientNet)	DeepFire (35 K images)	Up to 96.56	Cloud/desktop	Not optimized for edge deployment
CNN–RNN hybrid [5]	CNN + LSTM (temporal learning)	Mivia, Kaggle Fire	99.62/99.10	GPU workstation	Computationally intensive for real time
U-Net, Xception CNN [6,7,8]	Semantic segmentation	UAV and landslide datasets	76–92	UAV onboard GPU	Requires complex segmentation pipeline
Bonnet et al. [9]	IoT Visual + Audio ML (STM32 MCU)	Custom dataset	F1 = 96.0	STM32 MCU	Dual sensing increases complexity
TL with VGG16/ InceptionV3/Xception [10]	Transfer learning on mixed datasets	Satellite + Kaggle	Up to 98.72	Desktop GPU	Limited cross-dataset generalization
OPCNN [11]	Optimal CNN + J48 decision tree	Fire/non-fire (999 images)	95.11	CPU-based	Small dataset size
YOLOv4 [12]	Object detection (smart city)	27,600 images	99.8	GPU server	High computational demand
Deep + Weather fusion [13]	CNN + weather data integration	CCTV + weather	94.39	Centralized cloud	Depends on cloud connectivity
Hybrid CNN-LSTM [14]	CNN + LSTM (spatio-temporal)	Tomato leaf dataset	98.9	GPU	Not directly applied to fire detection
GAN + HOG + Adaboost + SVM [15]	Multi-level hybrid framework	Synthetic and real data	97.6	CPU-based	Synthetic data generation required
Adaboost-MLP + CNN [16]	Sensor and multimedia fusion	Environmental dataset	91.45	IoT node	Requires large heterogeneous data
FR + RF/SVM/LR [17]	Statistical hybrid (FR + ML)	Spatial data	AUC = 86.7–91.3	GIS environment	Manual feature selection needed
GMM + SVM/RF [18]	GMM-based video detection	Custom video data	89.97 (TPR), 21.7 FPS	Desktop CPU	Struggles with smoke occlusion
InceptionV3 cloud workflow [19]	Cloud CNN workflow	Satellite	91.0	Cloud infrastructure	Depends on connectivity
XGBoost + multisource fusion [20]	Remote sensing + ML fusion	Multi-sensor data	$R^{2}$ = 0.72	Remote sensing servers	Biomass estimation; not direct fire detection
FFDNet [21]	WSN-aided Xception + KELM	7000 images	99.02	Edge IoT nodes	Complex multi-module tuning

**Table 2:** Summary of dataset cleaning and duplicate removal.

Category	Original Count	After Cleaning
Fire	14,300	13,913
Smoke	14,300	13,823
Non-Fire	14,300	14,004
Total	42,900	41,740 (−2.7%)

**Table 3:** Classification report for the MobileNetV2 model on the test dataset.

Class	Precision	Recall	F1-Score
Smoke	0.98	0.99	0.99
Fire	0.99	0.97	0.98
Non-fire	0.98	0.98	0.98

**Table 4:** Ablation analysis of key components in the proposed MobileNetV2-based model.

Configuration	Accuracy (%)	Model Size (MB)	Latency (s/Frame)	FPS
Full Model (Two-Stage + Pruning + INT8 Quant.)	**97.98**	**3.4**	**0.77**	**1.30**
Without Two-Stage Training	96.84	3.4	0.78	1.28
Without Quantization	97.12	6.8	1.21	0.83
Without Pruning	97.45	4.6	0.89	1.12

**Table 5:** Performance comparison of the proposed MobileNetV2 model with existing lightweight architectures on the same dataset.

Model	Accuracy (%)	Macro-F1	AUC	Model Size (MB)	Latency (s/Frame)	FPS
ResNet18	96.24	0.962	0.995	11.8	1.21	0.83
EfficientNet-Lite0	97.12	0.972	0.996	5.9	0.92	1.09
MobileNetV3-Small	97.45	0.975	0.997	4.1	0.84	1.19
**Proposed MobileNetV2 (INT8)**	**97.98**	**0.983**	**0.998**	**3.4**	**0.77**	**1.30**

## Data Availability

The “Forest Fire Smoke and Non-Fire Image Dataset” used in this study is publicly available on Kaggle under the CC0: Public Domain license. The dataset can be accessed at: https://www.kaggle.com/datasets/amerzishminha/forest-fire-smoke-and-non-fire-image-dataset (accessed on 28 August 2025).

## References

[1] Lovejoy T.E., Nobre C., Nobre C. (2018). Amazon Tipping Point. Sci. Adv..

[2] Zaman M., Upadhyay D., Purcell R., Mutakabbir A., Sampalli S., Lung C.-H., Naik K. (2025). A Systematic Machine Learning Methodology for Enhancing Accuracy and Reducing Computational Complexity in Forest Fire Detection. Fire.

[3] Wang L., Zhang H., Zhang Y., Hu K., An K. (2023). A deep learning-based experiment on forest wildfire detection in machine vision course. IEEE Access.

[4] Khan A., Hassan B., Khan S., Ahmed R., Abuassba A. (2022). DeepFire: A novel dataset and deep transfer learning benchmark for forest fire detection. Mob. Inf. Syst..

[5] Ghosh R., Kumar A. (2022). A hybrid deep learning model by combining convolutional neural network and recurrent neural network to detect forest fire. Multimed. Tools Appl..

[6] Dewangan A., Pande Y., Braun H.-W., Vernon F., Perez I., Altintas I., Cottrell G.W., Nguyen M.H. (2022). FIgLib & SmokeyNet: Dataset and deep learning model for real-time wildland fire smoke detection. Remote Sens..

[7] Shamsoshoara A., Afghah F., Razi A., Zheng L., Fulé P.Z., Blasch E. (2021). Aerial imagery pile burn detection using deep learning: The FLAME dataset. Comput. Netw..

[8] Jin C., Wang T., Alhusaini N., Zhao S., Liu H., Xu K., Zhang J. (2023). Video fire detection methods based on deep learning: Datasets, methods, and future directions. Fire.

[9] Peruzzi G., Pozzebon A., Van Der Meer M. (2023). Fight fire with fire: Detecting forest fires with embedded machine learning models dealing with audio and images on low power IoT devices. Sensors.

[10] Sathishkumar V.E., Cho J., Subramanian M., Naren O.S. (2023). Forest fire and smoke detection using deep learning-based learning without forgetting. Fire Ecol..

[11] Jayasingh S.K., Swain S., Patra K.J., Gountia D. (2024). An experimental approach to detect forest fire using machine learning mathematical models and IoT. SN Comput. Sci..

[12] Avazov K., Mukhiddinov M., Makhmudov F., Cho Y.I. (2022). Fire detection method in smart city environments using a deep-learning-based approach. Electronics.

[13] Park M., Jeon Y., Bak J., Park S. (2022). Forest-fire response system using deep-learning-based approaches with CCTV images and weather data. IEEE Access.

[14] Shamta I., Demir B.E. (2024). Development of a deep learning-based surveillance system for forest fire detection and monitoring using UAV. PLoS ONE.

[15] Liu Z., Zhang K., Wang C., Huang S. (2020). Research on the identification method for the forest fire based on deep learning. Optik.

[16] Saeed F., Paul A., Hong W.H., Seo H. (2020). Machine learning based approach for multimedia surveillance during fire emergencies. Multimed. Tools Appl..

[17] Xu R., Lin H., Lu K., Cao L., Liu Y. (2021). A forest fire detection system based on ensemble learning. Forests.

[18] Wahyono, Harjoko A., Dharmawan A., Adhinata F.D., Kosala G., Jo K.-H. (2022). Real-time forest fire detection framework based on artificial intelligence using color probability model and motion feature analysis. Fire.

[19] Govil K., Welch M.L., Ball J.T., Pennypacker C.R. (2020). Preliminary results from a wildfire detection system using deep learning on remote camera images. Remote Sens..

[20] El-Madafri I., Peña M., Olmedo-Torre N. (2023). The wildfire dataset: Enhancing deep learning-based forest fire detection with a diverse evolving open-source dataset focused on data representativeness and a novel multi-task learning approach. Forests.

[21] Paidipati K.K., Kurangi C., Reddy A.S.K., Kadiravan G., Shah N.H. (2024). Wireless sensor network assisted automated forest fire detection using deep learning and computer vision model. Multimed. Tools Appl..

[22] Amerzishminha A. (2024). Forest Fire Smoke and Non-Fire Image Dataset. Kaggle. https://www.kaggle.com/datasets/amerzishminha/forest-fire-smoke-and-non-fire-image-dataset.

[23] Sandler M., Howard A., Zhu M., Zhmoginov A., Chen L.-C. MobileNetV2: Inverted Residuals and Linear Bottlenecks. Proceedings of the CVPR.

[24] (2024). Raspberry Pi Foundation Raspberry Pi 5 Specifications and Documentation. https://www.raspberrypi.com/products/raspberry-pi-5/.

